# 

**Published:** 1890-09

**Authors:** 


THE American Association of Obstetricians and Gynecologists will hold its next annual meeting in the City of Philadelphia, on Tuesday, Wednesday and Thursday, September 16, 17, and 18, 1890, in the hall of the College of Physicians, corner Thirteenth and Locust streets. All physicians interested are invited to attend the several sessions.

WM. WARREN POTTER, *Secretary*. E. E. MONTGOMERY, *President*.

---

THE next annual meeting of the Mississippi Valley Medical Association will be held in Louisville, October 8, 9, and 10, 1890. It is expected that this will be a large and interesting meeting. This Association is noted for the good work it accomplishes, and it may be predicted that, under the experienced leadership of Dr. Joseph M. Mathews, its president, the coming annual will be one of the best in its history.

### BOOKS AND PAMPHLETS RECEIVED.

Essentials of Anatomy and Manual of Practical Dissection, together with the Anatomy of the Viscera. Prepared especially for Students of Medicine. By Charles B. Nancrede, M. D., Professor of Surgery and of Clinical Surgery in the University of Michigan, Ann Arbor; late Senior Surgeon to the Episcopal Hospital; late Surgeon to the Jefferson Medical College Hospital; late Professor of General and Orthopedic Surgery in the Philadelphia Polyclinic, etc. Third edition, revised and enlarged, based upon the last edition of Gray's directory. Thirty handsome full-page lithographic plates in colors, and 180 fine wood-cuts. Small 8vo; pp. x.—388. Philadelphia: W. B. Saunders. 1890.

Essentials of Refraction and the Diseases of the Eye. By Edward Jackson, A. M., M. D., Professor of Diseases of the Eye in the Philadelphia Polyclinic and College for Graduates in Medicine, etc. Essentials of Diseases of the Nose and Throat. By E. Baldwin Gleason, S. B., M. D., Surgeon in Charge of the Nose, Throat and Ear Department of the Northern Dispensary of Philadelphia, formerly Assistant in the Nose and Throat Dispensary of the Hospital of the University of Pennsylvania, etc. Saunders's Question Compends No. 14, with 118 illustrations. 12mo; pp. viii.—276. Philadelphia: W. B. Saunders. 1890.

Railway Surgery. A Practical Work on the Special Department of Railway Surgery. For Railway Surgeons and Practitioners in the General Practice of Surgery. By C. B. Stemen, M. D., LL. D., Professor of Surgery in the Fort Wayne College of Medicine; Surgeon to the St. Joseph Hospital; Chief Surgeon of the Western Division of the

Pittsburgh, Fort Wayne & Chicago R. R., with numerous illustrations. 8vo; pp. 315. St. Louis: J. H. Chambers & Co. 1890.

Tenth Annual Report of the State Board of Health of New York for the Year 1889. Transmitted to the Legislature, February 20, 1890. 8vo, paper; pp. 532. Albany: James B. Lyon, State Printer. 1890.

Report of the Superintendent of Education of the City of Buffalo 1889-90. By James F. Crooker, Esq. Buffalo: Times Printing House. 1890.

The Pharmacology of the Newer Materia Medica, embracing the Botany, Chemistry, Pharmacy, and Therapeutics of New Remedies. Part I., Nov., 1889. Part II., Dec., 1889. Part III., Jan., 1890. Part IV., Feb., 1890. Part V., March, 1890. Detroit: George S. Davis. 1890.

I. University of the State of New York, Regents' Bulletin. No. 1. June, 1890. Laws, Ordinances and Forms.

II. Examination Department. Existing Laws Controlling the Practice of Medicine in the State of New York. Albany: University of New York. 1890.

Pierson-Sperling Lehrbuch der Electrotherapie. Fünfte Auflage bearbeitet von Dr. Med. A. Sperling, Berlin, mit 87 Abbildungen. Leipzig. Verlag von Ambr. Abel. 1890. Cloth, 12mo; pp. 355.

Price List and Subscription Catalogue of the Harderfold Hygienic Underwear. Manufactured and sold by the Harderfold Fabric Co., Troy, N. Y., U. S. A.

The Constitutional Treatment of Caries and Necrosis. By Hal. C. Wyman, M. S., M. D., Professor of the Principles and Practice of Surgery and Operative Surgery in the Michigan College of Medicine and Surgery. Detroit: Reprint from *The New York Medical Journal.*

The *Medical Calendar.* A Quarterly Compendium of Seasonable Therapeutics and Diatetics. Vol. I., No. 3. New York: Reed & Carnrick. 1890.

Dosimetry in Colorado. By J. E. McNeil, M. D. Reformation in the Practice of Medicine. By the same author. New York: W. J. Munson. 1890.

Cornell University, College of Agriculture. Bulletins of the Agricultural Experiment Station, Horticultural Division. XVIII. July, 1890. Experiences in Spraying Plants. XIX. Report upon the Condition of Fruit-Growing in Western New York, Ithaca, N. Y. 1890.

Report of the Buffalo Medical and Surgical Dispensary from January 1, 1889, to January 14, 1890.

Report of the Commission on New Asylum for Insane Criminals of the State of New York. Transmitted to the Legislature March 17, 1890 Albany: James B. Lyon, State Printer.

Cleanliness in Maternities. By Joseph Price, M. D., Physician to the Preston Retreat, Philadelphia. Reprint, *Medical and Surgical Reporter*, July 26, 1890.

A Case of Hematoma of the Ovary following Chronic Catarrhal Salpingitis, with Operation and Recovery. By R. Harvey Reed, M. D., Surgeon to the Baltimore & Ohio R. R., etc. Reprint, *Cincinnati Lancet-Clinic*, June 28, 1890.

Varicocele. By Thomas W. Kay, M. D., Scranton, Pa., ex-Surgeon to the Johanniter Hospital at Beyrout, Syria. Reprint, *Cleveland Medical Gazette*, December, 1889.

Report of One Year's Work of Intra-Pelvic Surgery for the Relief of Inflammatory Diseases. By Rufus B. Hall, M. D., Surgeon to the Cincinnati Free Hospital for Women, Professor of Gynecology, Cincinnati Polyclinic, etc. Reprint, *Lancet-Clinic*, June 21, 1890.

Report of a Case of Cholecystotomy, with Exhibition of Specimens. By Rufus B. Hall, M. D., Surgeon to the Cincinnati Free Hospital for Women ; Fellow of the American Association of Obstetricians and Gynecologists, etc. Reprint, *Times and Register*, July 5, 1890.

A New Operation for Prolapsus of the Vaginal Wall. By Andrew F. Currier, M. D. New York. Reprint, *Annals of Gynecology and Pediatry*. July, 1890.

A Successful Case of Nephrectomy. By George Ben. Johnston, M. D., Richmond, Va., late Professor of Anatomy, Medical College of Virginia, etc. Reprint, *Virginia Medical Monthly*.

Transactions of the American Dermatological Association at its Thirteenth Annual Meeting, held at Boston Medical Library, Boston, Mass., on the 17th, 18th and 19th of September, 1889. Reported by George H. Tilden, M. D., Secretary. New York : 1890.

The Limits of Vaginal Hysterectomy for Cancer of the Uterus. By Henry C. Coe, M, D. New York : Reprint *American Journal of Obstetrics*, June, 1890.

Coeliotomy : This, and not Laparatomy, is the Proper Greek Synonym of Abdominal Section, Laparatomy being an Incision of the Flank only. By Robert P. Harris, A. M., M. D., Philadelphia, Fellow of the College of Physicians, etc. Printed for the author by Wm. J. Dornan, Philadelphia. 1890.

The Use and Abuse of Soap and Water, By Merrill B. Ricketts, M. D., Professor of Dermatology and Syphilography Cincinnati Polyclinic. Reprint, *Journal of Cutaneous and Genito-Urinary Diseases*.

Cholecystotomy. By Edward Ricketts, M. D., Professor of Gynecology and Abdominal Surgery, Cincinnati Polyclinic, etc. Reprint, *The Pittsburg Medical Review*, May, 1890.

External Surgery of the Nose, by B. Merrill Ricketts, Ph. B., M. D., Professor of Dermatology and Syphilography, Cincinnati Polyclinic, etc. Reprint, *Journal American Medical Association*, June 11, 1890.

Functional Nervous Diseases of Reflex Origin. By Albert Rufus Baker, M. D., Professor of Ophthalmology in the Medical Department Wooster University, Cleveland, O. Reprint, *Journal American Medical Association*, May 21, 1890.

The New Treatment of Peritonitis. By Emory Lanphear, M. D., Kansas City, Surgeon to the East Side Dispensary, etc. Reprint, *Kansas City Medical Index*, July, 1890.

### HEALTH BULLETINS.

Michigan State Board of Health, August 7, 1890.

Newport, R. I., July, 1890.

New York State Board of Health, June, 1890.

Tennessee State Board of Health, August 20, 1890.

Abstract of Sanitary Reports, U. S. Marine Hospital Bureau, Washington, D. C. Vol. V., Nos. 31, 32, 33, and 34.

Restriction and Prevention of Diphtheria. Document issued by Michigan State Board of Health.

### MEDICAL COLLEGE ANNOUNCEMENTS FOR 1890-91.

University of Buffalo, Medical Department.

University of Buffalo, Department of Pharmacy.

University of Maryland, School of Medicine.

Syracuse University, College of Medicine.

Medico-Chirurgical College of Philadelphia.

Hahnemann Medical College and Hospital, Chicago, Ill.

---

Dentistry is to be taught as part of the surgical course in the medical colleges of Italy. Every one desiring to practice dentistry must hereafter take a medical degree.—*Coll. and Clin. Record.*

---

Ovariotomy is said to have been performed upon women many times before any experiment upon the lower animals was made.—*Ib.*

*Notice to Contributors.*—We are glad to receive contributions from every one who knows anything of interest to the profession. Articles designed for publication in the JOURNAL should be handed in before the first day of the month.

The Editors are not responsible for the views or opinions of contributors.

All communications should be addressed to

284 FRANKLIN STREET,
BUFFALO, N. Y.

